# African American women perceptions of physician trustworthiness: A factorial survey analysis of physician race, gender and age

**DOI:** 10.3934/publichealth.2018.2.122

**Published:** 2018-05-16

**Authors:** Jacqueline Wiltshire, Jeroan J. Allison, Roger Brown, Keith Elder

**Affiliations:** 1 Department of Health Policy and Management, College of Public Health, University of South Florida; 2 Department of Quantitative Health Sciences, University of Massachusetts Medical Center; 3 Research Design and Statistics Unit, School of Nursing, University of Wisconsin-Madison; 4 School of Public Health, Samford University

**Keywords:** trust in physicians, concordance, African Americans

## Abstract

**Background/Objective:**

Physical concordance between physicians and patients is advocated as a solution to improve trust and health outcomes for racial/ethnic minorities, but the empirical evidence is mixed. We assessed women's perceptions of physician trustworthiness based on physician physical characteristics and context of medical visit.

**Methods:**

A factorial survey design was used in which a community-based sample of 313 African American (AA) women aged 45+ years responded to vignettes of contrived medical visits (routine versus serious medical concern visit) where the physician's race/ethnicity, gender, and age were randomly manipulated. Eight physician profiles were generated. General linear mixed modeling was used to assess separately and as an index, trust items of fidelity, honesty, competence, confidentiality, and global trust. Trust scores were based on a scale of 1 to 5, with higher scores indicating higher trust. Mean scores and effect sizes (ES) were used to assess magnitude of trust ratings.

**Results:**

No significant differences were observed on the index of trust by physician profile characteristics or by medical visit context. However, the white-older-male was rated higher than the AA-older-female on fidelity (4.23 *vs.* 4.02; ES = 0.215, 95% CI: 0.001–0.431), competence (4.23 *vs.* 3.95; ES = 0.278, 95% CI: 0.062–0.494) and honesty (4.39 *vs.* 4.19, ES = 0.215, 95% CI: 0.001–0.431). The AA-older male was rated higher than the AA-older-female on competence (4.20 *vs.* 3.95; ES = 0.243, 95% CI: 0.022–0.464) and honesty (4.44 *vs.* 4.19; ES = 0.243, 95% CI: 0.022–0.464). The AA-young male was rated higher than AA-older-female on competence (4.16 *vs.* 3.95; ES = 0.205, 95% CI: 0.013–0.423).

**Conclusions:**

Concordance may hold no salience for some groups of older AA women with regards to perceived trustworthiness of a physician. Policies and programs that promote diversity in the healthcare workforce in order to reduce racial/ethnic disparities should emphasize cultural competency training for all physicians, which is important in understanding patients and to improving health outcomes.

## 1. Introduction

Trust is critical to the patient-provider relationship and is considered essential for effective health care delivery [1]. Patients' trust in their physicians is associated with satisfaction with care, information disclosure of sensitive information, adherence to treatments and continuity of care [1],[2]. African Americans are documented as one of the racial/ethnic minority populations with the lowest levels of trust in physicians [2],[3]. African Americans' low level of trust is frequently cited to explain their persistent and disproportionate burden of adverse health and healthcare outcomes [2],[4],[5]. It is also well documented that discrimination and prejudice/bias on the part of health care providers has perpetuated African Americans' low trust.

Research suggest that patient-provider race concordance (i.e., when patient and provider share the same race/ethnicity) may improve African Americans' trust and consequently their health outcomes [6],[7]. Patient-provider race concordance has been linked to higher trust, better communication, more shared medical decision making, higher satisfaction, greater use of medical care, and less perceived stigma and discrimination in the delivery of medical care [7]–[12]. African-Americans' have reported lower levels of trust with racially discordant providers [5],[13] and prefer providers of the same race/ethnicity [8],[12]. Nonetheless, reviews of the literature on patient-provider race concordance have concluded that strength of the evidence is modest in suggesting that ethnic minority patients would prefer and trust providers of similar racial backgrounds, or that patient-provider race-concordance is associated with more positive health outcomes [14]–[16].

Race/ethnicity is only one aspect of the human identity, which includes mutual aspects such as gender, age, and language [6]. Studies show that other types of concordance (including gender, age and language) concordance influence interpersonal care ratings of providers [6],[15],[17],[18]. Some patients have been shown to prefer doctors of the same gender [17],[18], with women more likely than men to prefer a provider of the same gender [18]. For example, a study of emergency room patients showed that women trusted female physicians more than male physicians and were more satisfied with their care [19]. The same pattern was not observed for men. Preferences and ratings have been linked to patient–physician communication and delivery of patient-centered care [20],[21]. However, the studies on patient preference and interpersonal ratings of care (which includes trust and satisfaction) neither included African Americans nor examined the mutual influence of race, gender, and age concordance on patients' preference and interpersonal ratings of care for physicians.

Understanding and eliminating racial/ethnic health disparities, particularly among African Americans, are amongst the most urgent problems facing our society today. Increasing the diversity of the healthcare workforce is proposed as a solution towards decreasing racial/ethnic disparities in health and healthcare [14],[15]. It is asserted that patients are more likely to choose physicians of similar racial/ethnic background when given the option [7],[9],[22]. But, it remains unclear whether African Americans would prefer a physician of similar racial/ethnic identity or how other aspects of the human identity (e.g., gender, age) may influence their preferences.

This study uses a factorial survey design based on social judgment theory [23] to assess older African American women's perceived trustworthiness of physicians based on manipulations of race/ethnicity, gender, and age of physicians. It also examines whether type of medical visit scenario influences perceptions of a physician's trustworthiness. Trust and concordance are complex concepts [6]. The factorial survey is specifically intended to clarify people's values and is a powerful technique for studying and analyzing people's choices and judgments about social phenomena that are complex or multidimensional in nature [24]. Older African American women constitute an expanding part of the elderly (and sickest) population in the United States [25]; hence, it is prudent to understand what influences older African Americans' trust in providers given its importance in adherence, disease management and healthy aging [26].

## 2. Methods

### 2.1. Study Design and Procedure

This study utilizes the factorial survey technique, an approach which combines experimental design and survey research methods where short descriptions of situations or persons (i.e., vignettes) are provided to participants within surveys in order to elicit their opinions or judgments about each vignette provided [23],[27],[28]. People routinely make judgments with consideration of various factors. The factorial survey is effective in identifying variability and patterns associated with factors used in making judgments. The factorial survey approach has been used to assess multidimensional phenomena such as preference for shared decision making in the medical encounter, preferences for racial integration, and racial/ethnic childcare preferences [29],[30].

In our factorial survey, participants are presented with two vignettes of an initial physician visit: one vignette describes a routine medical checkup with no serious concerns, while the other vignette describes a visit in which the woman had felt a lump in her breast. Each vignette is accompanied with a photograph of an imaginary physician and participants are asked to judge the physician on perceived fidelity, competence, honesty, confidentiality, and global trust. The race/ethnicity, age, and gender of the imaginary physicians were randomly manipulated. This was a factorial balanced incomplete design; women were not exposed to all possible imaginary physicians. Each woman was given four imaginary physicians to evaluate. Women were given the routine visit vignette twice with two different imaginary physicians and the serious health concern visit vignette twice with two different imaginary physicians. Women had the choice of listening to or reading the vignette on the computer. Pictures of the imaginary physician and vignette were linked to a response instrument in the simulation.

Thirty-two individuals were recruited to be used in pictures as imaginary physicians. Each individual wore a white lab coat and stethoscope and was photographed against a blue background. Eight photographs were selected to use in the survey. The photograph selection was based on the highest averaged score of attractiveness, likability, and professionalism from six African American women (aged 45 and over) in the community. Seven items from the Reysen Likeability scale was used to assess, rank, and select physicians [31].

### 2.2. Visit Scenario

The following is the introduction script for the serious medical visit that participants received before being presented with the picture of the imaginary physician and trust question. The non-serious or routine visit does not include the statement “You are very concerned about this visit because you (think you have a lump in your breast or) think you felt a lump in your breast.”

You have been a patient at the community clinic for one year. The practice has a good reputation and you like the care that you have received. However, you do not always see the same doctor at every visit. You are scheduled to see a doctor today for an annual physical exam, which will include a breast exam. You are very concerned about this visit because you (think you have a lump in your breast or) think you felt a lump in your breast. The receptionist greets you and asks you to update any personal information in your record. You wait approximately 15 minutes; then the nurse takes you back to an examination room. The nurse reviews some information regarding your past medical history. Your blood pressure, temperature, and pulse are taken, and then you are asked to undress from your waist up and put on a gown. The next person to greet you is this doctor…

### 2.3. Study Sample

A convenience sample of 313 African American women aged 45–64 years residing in a Midwestern city was recruited. African Americans are a difficult population to recruit into epidemiologic studies [32], therefore, women were recruited actively through churches, community health centers, hair salons, health fairs, community events, senior centers and housing establishments, and through advertisements in the African American community. Women were also recruited through referral by other study participants. While these community-based recruitment methods may compromise sample representativeness, these methods have proved successful in recruiting African Americans into epidemiologic studies [32]. The sample size required for this survey was calculated on the assumption that African American women would have an average score of 3.98 on the trust measures for White physicians (with a standard deviation of 1.00), and projected that they would have 5% increase in trust when exposed to African American physicians. These sampling criteria are based on a statistical power of approximately 0.8 with an alpha = 0.05 [33].

The factorial survey was administered via laptops in university and community settings. Older and disabled women were surveyed in their homes (n = 40). Participants were also assisted with the reading of the survey and laptop technology was used when needed. Participants received a $20 gift certificate for completing the surveys. This study was approved by the University of Wisconsin-Madison Social and Behavioral Science Institutional Review Board.

### 2.4. Dependent Variables

Judgments about physicians were made based on five measures of trust (i.e., fidelity, competence, honesty, confidentiality, and global trust) from the Wake Forest physician trust scale [34]. Ratings range from 1 to 5 (strongly agree to strongly disagree). Trust measures are as follows: *I think this doctor will do whatever it takes to get me all the care that I need* (fidelity), *I think this doctor will be totally honest about the care I need* (honesty), *I think this doctor will keep all my information private* (confidentiality), *I think this doctor will be extremely thorough and careful* (competence), and *I think I can trust this doctor to put my medical needs above all other considerations when treating my medical problems* (global trust). The five items were assessed separately and as an index (i.e., summed and averaged) of trust in each doctor [35].

### 2.5. Independent Variables

Profile characteristics of imaginary physicians: Women were presented with pictures which portrayed the following profile characteristics of physicians: African American young male (AAYM), African American older male (AAOM), African American young female (AAYF), African American older female (AAOF), White young male (WYM), White older male (WOM), White young female (WYF), and White older female (WOF). Young was categorized as age 30–45 years and older as 50+ years.

Respondents' characteristics: Covariates included in our analysis are based on conceptual and empirical literature indicating that trust in doctors is related to patient sociodemographic characteristics, health status, access to care, and medical care factors [1]. Covariates included as confounders were: age (45–64, 65+), education level (less than high school, high school, some college, college and above), income level (<$25,000, $25,000–$49,999, $50,000–$74,999, ≥$75,000), insurance status (insured, uninsured) perceived health status (poor/fair, good, and very good/excellent), usual source of care (yes/no), and negative experience with a health provider (yes/no).

### 2.6. Analysis

Subjects' perceptions of trust in the 8 vignettes were analyzed using factorial survey methods [36], a technique for applying experimental design to survey research. This method is used primarily to study participants' responses to hypothetical situations, such as manipulated vignettes. MLWin software [37] was used to construct a two-level hierarchical linear model (vignettes nested within participants). This approach poses two regression models simultaneously: one modeling the vignette and scenario effects within the subjects, and one modeling the subject effect between subjects. This approach allows the partitioning of the total dispersion in the estimated regression parameters into a sampling variance and a residual variance. Through simultaneous use of information from individual respondents and the entire group of respondents, hierarchical models produce more precise parameter estimates than other methods [36]. Other analytic approaches, such as a single-level analysis and a separate-level, two-stage analysis were not used, because their assumptions are violated by factorial survey designs.

The hierarchical linear analysis was conducted on vignette variables and the severity scenario condition using multiple outcome measures of trust. Seven dummy variables (k-1 dummies) were used to represent the different vignette conditions, using the African American older female vignette as the reference group. Another dummy variable was used to represent the severity level of the scenario, with the non-serious scenario used as reference. Parameter estimates were obtained by an iterative generalized least-squares estimator. All vignette variables were included in the within-respondents portion of the model, and crossed with the severity scenario condition. All subject characteristics (age, education, income, health status, insurance coverage, usual doctor, and negative experiences encountered) were also incorporated into the subject's intercept in the between-subject portion of the model (See the Appendix in Brown, Brown, Edwards, and Nutz, 1992 for more details) [38]. Means, p-values, effect size estimations (Cohen's *d*) with 95% confidence intervals were used to assess effects. Results were considered statistically significant at *p*-value < 0.05. The GLMM models were constructed in NCSS Version 7 [39].

## 3. Results

Characteristics of the study participants are presented in Table 1. Approximately 27% of the women were elderly (65+ years), 18.2% had less than a high school diploma, 6.4% had no usual source of care, 45.7% had an income of <$25,000, 16% had no health insurance, and 44% reported having had a negative experience with the health care system. The unadjusted overall mean scores were higher for confidentiality and honesty. Scores are based on a scale of 1 to 5, with higher scores indicating higher trust. While results from the bivariate analysis are not shown, the white-older-male had a significantly higher unadjusted mean score than the African American older female (AA-older-female) on competence (3.94, 3.71; *p* = 0.034) and the AA-older-male had a significantly higher unadjusted mean score than the AA-older-female on honesty (4.08, 3.86; *p* = 0.049). The AA-older-female was no different than counterparts on fidelity, confidentiality, global trust, or overall (i.e., index of trust) trust.

**Figure 1.**
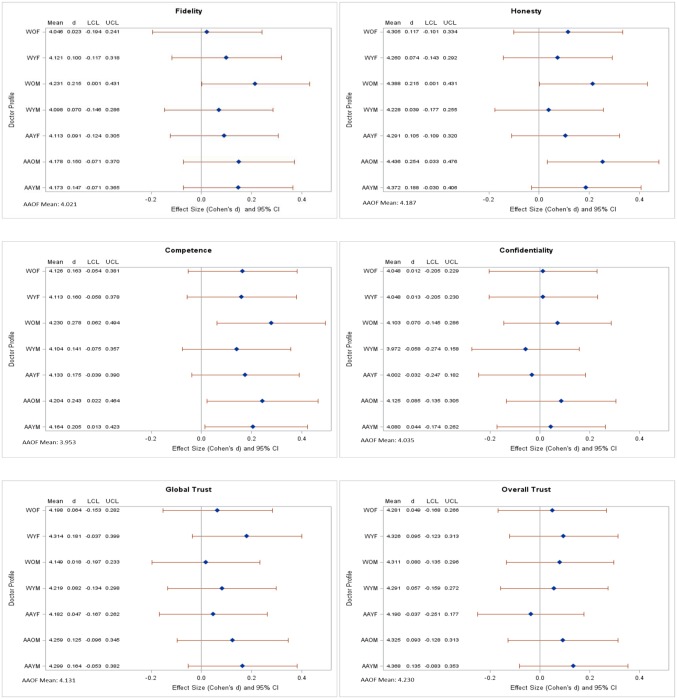
Adjusted Means and Effect Size Estimations with 95% Confidence Intervals for Trust Measures by Physician Profile Characteristics*†.

Figure 1 shows the adjusted mean (*x*) scores and the effect size estimations with confidence intervals from the hierarchical linear analyses assessing (1) the effects of physician profile characteristics (i.e., race, age, and gender) on African American women's perception of physician trustworthiness and (2) severity of visit (routine versus serious health concern visit) on women's perceptions of physician trustworthiness. After adjusting for all study covariates, there were no significant differences in overall (or index of) trust by physician profile characteristics. However, there were significant differences by physician profile characteristics on the individual measures of fidelity, competence and honesty. White-older-male when compared to AA-older-female were rated more favorably on fidelity (4.23, 4.02; ES = 0.215, 95% CI: 0.001–0.431), competence (4.23, 3.95; ES = 0.278, 95% CI: 0.062–0.494) and honesty (4.39, 4.19, ES = 0.215, 95% CI: 0.001–0.431). AA-older-male were rated more favorably than AA-older-female on competence (4.20, 3.95; ES = 0.243, 95% CI: 0.022–0.464) and honesty (4.44, 4.19; ES = 0.243, 95% CI: 0.022–0.464). AA-young-male was rated more favorably than AA-older-female on competence (4.16, 3.95; ES = 0.205, 95% CI: 0.013–0.423). There were no significant differences in perceptions of trust by type of medical visit (results not shown). Higher education was associated with less favorable scores on fidelity, honesty, global trust, and overall trust index (results not shown).

**Table 1.** Characteristics of Study Sample (N = 313).

	% (N) or mean (SD)
Age	
45–64	73.2 (229)
65+	26.8 (84)
Education	
< High school	18.2 (57)
High school	23.0 (72)
Some college	31.0 (97)
College +	27.8 (87)
Income	
< $25,000	45.7 (143)
$25,000–$49, 999	30.7 (96)
$50,000–$74, 999	13.4 (42)
$75,000 or more	10.2 (32)
No health insurance	16.0 (50)
No Usual provider	6.4 (20)
Self-reported health	
Poor	7.4 (23)
Fair	24.3 (76)
Good	43.7 (137)
Very good	20.4 (64)
Excellent	4.2 (13)
Negative experience with health provider	43.8 (176)
Trust measures*	
Fidelity	3.82 (1.004)
Competence	3.86 (0.986)
Honesty	3.96 (0.993)
Confidentiality	4.07 (1.012)
Global trust	3.85 (1.041)
Trust index score†	3.91 (1.007)

*Scores are based on a scale of 1 to 5, with higher scores indicating higher trust.

^†^Five trust measures were summed and averaged.

## 4. Discussion

In this study, African American women were given medical visit vignettes and pictures of imaginary pictures physicians (in which race/ethnicity, age, and gender were randomly manipulated) and asked to make judgments about perceived fidelity, competence, honesty, confidentiality, and global trust of the physician. The index of trust (five measures were summed and averaged) did not differ significantly by physician characteristics. However, there were small statistically significant differences by physician characteristics on the measures of fidelity, honesty, and competence. The White-older-male was rated more favorably than the AA-older-female on fidelity. The White-older-male and the AA-older-male were rated more favorably than the AA-older-female on honesty. The White-older-male, AA-older-male, and AA-young-male were rated more favorably than AA-older-female on competence. Type of medical visit did not influence perceptions of physician trustworthiness.

Our finding of no difference in overall trust by physician characteristics suggest that our sample of African Americans may not have physician preferences or it may be that they had professed overall trust for physicians irrespective of race/ethnicity, gender and age. This is largely consistent with the reviews on patient-physician race concordance, which concluded that the majority of patients have no preference regarding providers' race/ethnicity and are very satisfied with the care they receive from physicians of dissimilar race/ethnicity [14],[15]. However, this finding is more in line with Street and colleagues (2008) study which showed that patients perceptions of shared identity of race, ethnicity, and community with physicians was not associated with trust; whereas perceptions of shared personal believe, values and ways of communicating were associated with higher ratings of trust in physicians [6]. Street and colleagues (2008) also found that some patients in race and gender concordant dyads perceive themselves dissimilar from their physicians, while others in discordant dyads perceive themselves as similar to their physicians [6]. On balance, these data represent aggregates; individual differences are bound to exist.

On the other hand, our slightly higher ratings for older male physicians compared to older female African American physicians on selected measures of trust may refer to medical setting, societal beliefs, patient values and expectations [1],[40]. Kumar and colleagues (2009) found that race concordance or discordance with a physician was largely a function of the medical setting [41]. In settings that employ a large number of African American physicians, Whites with health insurance are more likely to be in concordant dyads than Whites without insurance and African Americans without insurance were more likely to be in a concordant dyad than African Americans with insurance [41]. Race concordant or discordant dyads did not influence patients' perceived quality of care [41]. There are situations, context, and settings that shape patients' perceptions of providers and healthcare; thus, it is critical that we do not over generalize the importance of concordance to patients.

Gender roles and beliefs are pervasive in society, in many professional contexts women's work roles and competences are more likely to be devalued [42]. Gender differences favoring men also exist in patients' evaluation of clinical performance: many patients do not give female physicians the credit they deserve [40]. Research shows that high verbal patient-centered behavior is regarded as an indicator of clinical competence for male physicians but not female physicians [21]. Female physicians have a more patient-centered practice style which is preferred by patients and is associated with higher patient satisfaction [40],[43]. However, the meta-analysis of patient satisfaction and physician gender conducted by Hall and colleagues found an almost nonexistent difference favoring female physicians on care satisfaction [43]. Female physicians were slightly favored when physicians were less experienced when satisfaction pertained to a specific visit, and when patients were young [43]. Relatedly, the slight preference for older male physicians (on fidelity, honesty, and competence) by the older African American women in our study may be due to implicit or unconscious bias [21].

We did not demonstrate a relationship between physician characteristics and perceived trustworthiness of physicians, which may speak to the complexity of concordance, trust and the patient-provider relationships that have been documented [1],[6],[14]. A gestalt of patient, provider and system factors may determine patient trust and preference for concordance [1],[14],[15]. While concordance may orient patients and physicians towards some common ground [6], a physician's character and personality in an ongoing patient-physician relationship impacts a patient's trust in that physician [1].

Our results should be considered in the context of several limitations. First, we used a convenience sample of women which precludes generalizability to other African American populations. Second, there is evidence of discrepancies between social judgment in vignette surveys and actual behavior [27]. Third, every aspect of a social phenomenon cannot be simulated and it is also difficult to assess the extent to which social desirability influenced the subjects' responses [24]. Finally, this study used observational methods and therefore cannot define cause-and-effect associations. However, with the paucity of research on trust among older African Americans [26] and the ambiguity regarding patient preferences for concordance with their physicians [14],[15], this research fills a gap in the literature and takes a novel approach to understanding how older African American women may make choices and judgments about trusting their physicians.

Our study has implications for programs and policies focused on reducing racial/ethnic disparities by promoting diversity in the healthcare workforce. Our findings suggest that concordance may hold no salience for some groups of older African women. The relevance of race/ethnic concordance has been questioned because it does not account for multiracial identity and the variability in values, beliefs, and culture within racial/ethnic groups [14]. Moreover, it might create the perception that health equity can only be achieved in race-concordant patient-physician, which is unrealistic given our multiracial identity and cultures [14]. Promoting diversity in the medical profession is a laudable goal that will address an equity issue [44]; however, we should also strive to create a culturally competency diverse healthcare workforce. While diversity may reduce disparities in access because ethnic minority physicians are more likely to serve disadvantaged populations [15], training culturally competent physicians is paramount in reducing disparities in health outcomes [6],[15],[44]. Cultural competency can foster trust and communication, which are critical to the patient-provider relationship and to improving disparities in health outcomes [1],[6],[15]. Cultural competency training can transcend concordance issues to establish a positive relationship with a patient, which contributes to positive health outcomes [6].

## Footnotes

* Doctor Profile Abbreviations: AAYM: African American Young Male; AAOM: African American Older Male; AAYF: African American Young Female; AAOF: African American Older Female; WYM: White Young Male; WOM: White Older Male; WYF: White Young Female; WOF: White Older Female.

† All profiles were compared to AAOF. Effect size: d = 0.1 (small effect), d = 0.3 (medium effect), d = 0.5 (large effect).
